# Management of Pepper Weevil (*Anthonomus eugenii* Cano) (Coleoptera: Curculionidae) Using Biorational and Conventional Insecticides in South Florida

**DOI:** 10.3390/insects16111101

**Published:** 2025-10-29

**Authors:** Victoria Adeleye, Dakshina Seal, Oscar Liburd

**Affiliations:** 1 Tropical Research and Education Center, University of Florida, IFAS, Homestead, FL 33031, USA; adevicky14@gmail.com; 2 Entomology and Nematology Department, University of Florida, Gainesville, FL 32611, USA; oeliburd@ufl.edu

**Keywords:** pepper weevil, conventional insecticides, isocycloseram bio-rational insecticides, insecticide rotation, adult suppression, infested fruits, marketable yield

## Abstract

**Simple Summary:**

Newer insecticides with various modes of action are required to rotate with the already available and effective insecticides for successful pest management. This research sought to identify effective insecticides that can be used in rotation with the few effective ones that are already available for the management of pepper weevil in South Florida. Isocycloseram was found to be effective in several studies and can be rotated with thiamethoxam and Vydate for effective control.

**Abstract:**

In five studies, we determined the potential of biorational and conventional agrichemical insecticides for controlling pepper weevil in pepper in small field plots arranged in a randomized complete block design replicated four times. Insecticide treatments were applied alone or in a program. The first study (spring 2019 Mar–Jun) evaluated the efficacy of five conventional insecticides applied alone or in rotation. All conventional insecticides significantly reduced pepper weevil-infested fruit (0–0.40 fruits/treatment) as compared to the untreated control (0.95–2.0)/treatment) but did not differ from the check in the mean marketable yield. In the second (spring 2021 Mar–Jun) and third (spring 2022 Feb–May) studies, proper placement of isocycloseram in four different rotation programs with oxamyl and thiamethoxam provided significant reduction of pepper weevil adults (0–0.2 adults/insecticide treatments, 1.1 adults/control plot) and infested pepper fruit, and an increase in marketable yield. In the fourth (spring 2022 Mar–Jun) and fifth (Dec 2022–Mar 2023) studies using two conventional and two biorational insecticides, *Beauveria bassiana* strains boteGHA significantly reduced adults and infested fruits and was comparable with thiamethoxam and isocycloseram. Isocycloseram significantly increased marketable yield (34,500 kg/ha). The novel insecticide, isocycloseram, is an effective alternative that can be used in rotation with the already available insecticides for pepper weevil management.

## 1. Introduction

The pepper weevil is the primary pest of peppers throughout the southern United States. Its range extends from tropical and subtropical regions of North America, Central America, and the Caribbean [1,2,3,4,5,6]. The pepper weevil was first reported in Mexico in 1894 [7], and in Texas, USA, in 1904; California in 1923; Hawaii in 1933; Florida in 1935; Canada in 1992; Virginia in 2007; and the Netherlands in 2012 [4,7,8,9]. Van De Vossenberg et al. [10] suggested that Mexico is likely the origin of the pepper weevil. Pepper weevil was detected and successfully eradicated from fields in Italy [11] and greenhouses in western Canada [12] and the Netherlands [9]. Pepper weevil infestation was observed in both fields and greenhouses in southern Ontario, Canada [13,14].

The major management tool used by growers for controlling pepper weevil is the use of broad-spectrum insecticides [15,16,17]. However, repeated and heavy use of insecticides has led to the development of resistance in pepper weevils, an increase in environmental contamination, and a reduction in natural enemy populations [1,18]. Andrews et al. [1] compared the difference between manual collection and destruction of fallen infested fruits with chemical control techniques. They found that visual scouting and the application of toxaphene to control adults was more cost-effective than removing and destroying fallen fruits. Eller et al. [19] considered this technique to be risky and time-consuming because there is a possibility that weevils are detected before the action threshold level. This could result in an untimely insecticide application.

A reduced-risk insecticide-based management approach was also evaluated recently in Canada, where conventional, microbial, and reduced-risk insecticides were used in laboratory bioassays and greenhouse trials against pepper weevil. Eight out of 16 insecticides tested were effective and were further used in greenhouse cage trials [20]. These reduced-risk insecticides can be used in IPM programs, but further research is needed to evaluate their compatibility with biological control agents.

The efficacy of biorational (azadirachtin and spinetoram) and conventional insecticides (thiamethoxam, chlorpyrifos, malathion, and λ-cyhalothrin) was evaluated against pepper weevil in a jalapeño pepper field in South-Central Chihuahua. Thiamethoxam, chlorpyrifos, and λ-cyhalothrin significantly controlled pepper weevil at 0, 3, and 5 days after application in the pepper field. They recommended the rotation of spinetoram, chlorpyrifos, and thiamethoxam for pepper weevil management [17].

The immature life stages cause damage and are found inside the fruit [14]. This makes only the adult stage susceptible to insecticides for control [14]. Most of the insecticides used for the management of pepper weevil are carbamates, organophosphates, pyrethroids, neonicotinoids, and ryanoids [17,18,21,22,23]. Pepper weevil resistance to some of the insecticides in the above-mentioned groups has been reported. An example is the case of resistance to carbamate insecticides, including carbaryl and methomyl, in one of the three pepper weevil populations evaluated in a study [18]. In a bioassay using the topical application method, the pepper weevil populations evaluated were more tolerant to thiamethoxam, followed by oxamyl and malathion [24].

Thiamethoxam^®^ and Vydate^®^ are standard insecticides used in rotation for the control of pepper weevil. These insecticides have the potential to double the marketable yield compared with the control [25]. To delay the development of resistance, it is recommended to use different insecticides with different modes of action in a rotation program [15]. Therefore, this research aims to determine the efficacy of novel and biorational insecticides that can be rotated with the already available conventional insecticides. This will help reduce the fast development of resistance and give other alternatives to the two major insecticides used for managing pepper weevil in South Florida.

## 2. Materials and Methods

### Study Area and Field Preparation

These research studies were conducted in the research plots located in the Tropical Research and Education Center (25.513° N, −80.504° W), University of Florida-IFAS, Homestead, FL, USA. The soil type of all plots was in the Rockdale class, comprising 70% limestone pebbles. The soil was plowed (CASE IH agriculture) and disked (Athens Plow Co. Inc., Athens, TN, USA). Pepper plants in all studies were set on raised beds of 91 cm wide and 15 cm high with 1.83 m between centers and were prepared with Kennco superbedders (Kennco Manufacturing Co. Inc., Atoka, OK, USA). The raised beds were covered with white-on-black plastic mulch (Canslit Inc., Victoriaville, QC, Canada, and supplied by IMAFLEX USA Inc., Thomasville, NC, USA). Prior to mulch placement, granular fertilizer (N-P-K: 6-12-12) was broadcast on the bed soil at the rate of 1344 kg/ha and was incorporated within 2–4 inches of the soil surface. For irrigation, two drip tubes having emitters spaced 30 cm apart were placed at the center with 15 cm spacing at the time of laying down plastic.

Jalapeño pepper transplants of six-week-old were planted in the bed with 12-inch spacing within the bed and six feet center to center in between beds. Plants were irrigated twice weekly, one hour each time, delivering 1.0 in each time. *Bacillus thuringiensis*-based insecticide was applied to control occasional infestation of the beet armyworm (*Spodoptera exigua* Hubner). Various conventional and biorational insecticides were evaluated to control pepper weevil by using insecticides alone or in a program. We conducted five studies in four years, 2019–2023. In all studies, insecticide treatments were applied through foliar application and were initiated at first bloom and continued for four to six dates using a manually operated backpack sprayer with two nozzles delivering 30 GPA at 30 psi.

In the first study conducted in spring 2019 (Jan–Apr), the efficacy of the insecticides spirotetramat (Movento^®^) and flupyradifurone (FPF, Sivanto^®^) was evaluated against pepper weevil on jalapeño pepper and compared with a local standard conventional insecticide, thiamethoxam (Actara^®^). Spirotetramat acts as a lipid biosynthesis inhibitor [26,27]. It is ambimobile, having the chemical formula C_21_H_27_NO_5_, which moves upward and downward through the vascular system. Spirotetramat significantly reduces females’ fecundity and fertility. Flupyradifurone is a butanolide insecticide. Flupyradifurone acts as a reversible agonist on insect nAchR. It has high translaminar activity against the hidden pest [28]. Thiamethoxam is a broad-spectrum second-generation neonicotinoid insecticide. It works agonistically on insect nicotinic receptors (nAChR) [29]. The experiment was conducted in spring 2019 at the Tropical Research and Education Center (TREC) (25.511° N, −80.501° W). The experimental design was a randomized complete block design with four replications. The treatment consisted of 2 beds, each 20 ft long and 6 ft wide with a 5 ft buffer. Five treatments, including control, were evaluated in the study (Table 1).

In the second study conducted in spring 2021 (Mar–Jun), where insecticides were used in rotation programs, the efficacy of the novel insecticide isocycloseram (Plinazolin^®^, IRAC Group 30) was evaluated and compared with growers’ local standard insecticides, thiamethoxam (Actara^®^, IRAC Group 4A) and oxamyl (Vydate L^®^, IRAC Group 1A). Isocycloseram is a novel isooxazoline insecticide that is characterized as a non-competitive GABA-gated chloride channel antagonist [30]. Oxamyl is a carbamate pesticide that has the chemical formula C_7_H_13_N_3_O_3_S. It is an acetylcholinesterase inhibitor and neurotoxicant. The optimal placement of isocycloseram was determined in a program approach for pepper weevil control on jalapeño pepper.

Five treatments of various modes of action were evaluated in this study (Table 2), which included: i. untreated control, ii. isocycloseram applied on the first and second spray dates, followed by a rotation between thiamethoxam and oxamyl in the remaining 4 weeks, iii. isocycloseram applied on the fifth and sixth spray dates, thiamethoxam applied on the first and third spray dates, oxamyl on the second and fourth spray dates, iv. isocycloseram applied on the third and fourth spray dates, thiamethoxam on the first and fifth spray dates, and oxamyl on the second and sixth spray dates, v. thiamethoxam applied on the first, third, and fifth spray dates while oxamyl applied on the second, fourth, and sixth spray dates (Table 3).

The third study is a repetition of the second study and was conducted in spring 2022 (Feb–May). All materials and methods were the same as in the second study. Six weekly applications were made in the second and third studies.

In the fourth study conducted in spring 2022 (Mar–Jun), the efficacy of biorational insecticide (Velifer, BoteGHA^®^) and the conventional insecticide (isocycloseram) was evaluated against pepper weevil on jalapeño pepper and compared with a local standard conventional insecticide (thiamethoxam) (Table 2). Velifer is a naturally occurring beneficial fungus, *Beauveria bassiana* strain PPRI 5339. BoteGHA is an organic bioinsecticide consisting of *Beauveria bassiana* strain GHA. Both fungal strains cause death to the insects by the germination of fungal spores inside the insect bodies. This study evaluated the level of control provided by the insecticide isocycloseram as well as the biorational insecticides. An adjuvant, Dyne-Amic, a blend of methylated seed oil (MSO) + organosilicone-based surfactants, was added to all insecticides tested at the appropriate commercial label rate.

The fifth study is a repetition of the fourth study conducted in spring 2023 (Dec 2022–Mar 2023). Six applications were made for all insecticides except for the BoteGHA, which was applied every 4 days, totaling 10 applications. Weekly sampling was completed for 6 weeks. Five treatments, including control, were evaluated in the study (Table 2). The treatment plots in studies 2–5 consisted of beds that were 20 ft long and 6 ft wide, with a 5 ft buffer.

## 3. Experimental Design, Insecticide Application, and Evaluation of Treatments

The experimental design employed a randomized complete block design with four replications in all studies. Five treatments, including control, were evaluated in all studies. Local agronomic practices were followed to ensure the pepper plants were in optimal condition. The method of land preparation, treatment application, and evaluation of treatments was the same as explained in the previous study by Adeleye et al. [31]. Crop injury was visually assessed for phytotoxicity after isocycloseram application and compared with the control treatments. Phytotoxicity ratings ranged from 0 to 100%. When no phytotoxicity was observed, data were recorded as zero, and complete death of plants represents 100%.

In all studies, to evaluate the efficacy of the insecticide treatments, 48 h after each spray application of treatment products, the number of fallen infested fruit on the bed surface was manually collected and counted from each treatment plot. Five plants from each treatment were visually checked to count the number of adults on pepper foliage, except in the second study. The number of marketable fruit from each plot was counted and weighed using a Pelouze scale (Pelouze Manufacturing Company, 232 E. Ohio St., Chicago, IL, USA) at the end of the season.

Compadre Syngenta pepper seedlings were used in 2021. Seminis jalapeño seeds and Syngenta’s compadre seeds were used in the 2022 and 2023 studies, respectively. Seeds were sown in Promix soil (Premier Tech Horticulture Inc., 200 Kelly Rd., Unit E-1, Quakertown, PA 18951, USA) in seedling trays in a greenhouse and transplanted in the field after six weeks.

Statistical Analysis. Response variables include adults on plants, infested fruit counts, and marketable yield. In the first study, data were analyzed using PROC GLM (SAS version 9.4), and the means were separated using the Waller–Duncan K-ratio *t*-test. In the second, third, fourth, and fifth studies, data were analyzed using a mixed model ANOVA (PROC GLIMMIX model, SAS). The pepper weevil population was normally distributed in the study area, where individual data points varied within a short range, showing a short-tailed right skew. To normalize error variance, data were square root transformed to normalize error variances before analysis. The treatments and blocks were considered as fixed and random effects in the model, respectively. The non-transformed means and standard errors were reported in the table. Tukey’s HSD procedure was used to determine differences amongst means, and all data were analyzed at a 5% significance level.

## 4. Results

### 4.1. Treatment Effect on Pepper Weevil Adults on Foliage

When data from the first study (spring 2019, Mar–Jun) were pooled across sampling dates, there were no significant differences among treatments. However, thiamethoxam, followed by spirotetramat-treated plants, had a numerically lower number of adults compared to the rest of the treatments. No adult was found on March 21. Adult counts in the insecticide-treated plots on March 21 and March 29 did not differ from the untreated control. On April 4, all treatments significantly reduced PW adults as compared to the untreated control. On April 12, April 19, and April 2, insecticide treatments did not reduce PW adults when compared with the untreated control (Table 4).

**Figure 1:**
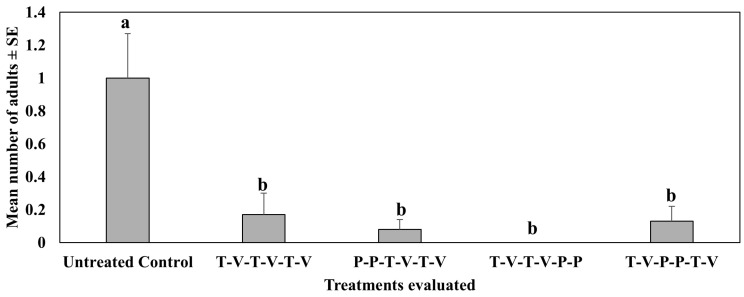
Mean number of adults ± SE during the 6-week sampling period in the third study, spring 2022 (Feb–May). See Table 3 for the rotation schedule. T: Thiamethoxam, V: Vydate, P: Isocycloseram. Means with the same letter are not statistically significant.

The second study (spring 2021, Mar–Jun) did not evaluate the number of adults on plants. In the third study, all insecticide treatments used in rotation significantly reduced, at least ca. 20% each, pepper weevil adults on the foliage as compared to the untreated control plants (Figure 1). Untreated control plants had at least five times more adults than any of the insecticide treatments. Plants treated with thiamethoxam followed by oxamyl at weekly intervals for four weeks and then followed by two back-to-back applications of isocycloseram on the fifth and sixth weeks did not have any pepper weevil adults, although this was not statistically different from other insecticide treatments. All rotation treatments with isocycloseram, at the beginning or the end, have numerically fewer adults on foliage than the treatment without isocycloseram or otherwise. There were significant differences across dates (F_5,87_ = 8.20, *p* < 0.0001) and across treatments (F_4,87_ = 15.91, *p* < 0.001), and there were significant interactions between dates and treatments (F_20,87_ = 2.62, *p* = 0.0011).

**Figure 2:**
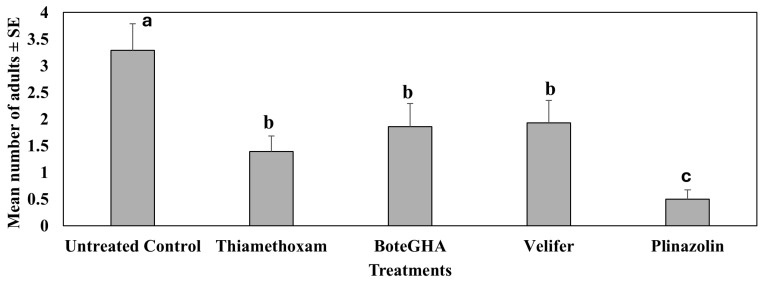
Mean number of adults on plants ± SE during the 6-week sampling period in the fourth study, spring 2022 (Mar–Jun). Means with the same letter are not statistically different according to Tukey’s HSD Test at *p* < 0.05.

In the fourth study conducted in spring 2022 (Mar–Jun), biorational insecticides containing *Beauveria bassiana* strain PPRI 5339 (Velifer) and *Beauveria bassiana* strain GHA (boteGHA) significantly reduced (ca. 57.14%) pepper weevil adults as compared to the untreated control (Figure 2).

These two biorational insecticides did not differ statistically from the thiamethoxam-treated plants in the mean number of adults. In this study, isocycloseram (Plinazolin) was superior in reducing pepper weevil adults by about 85.71% in comparison to the untreated control.

**Figure 3:**
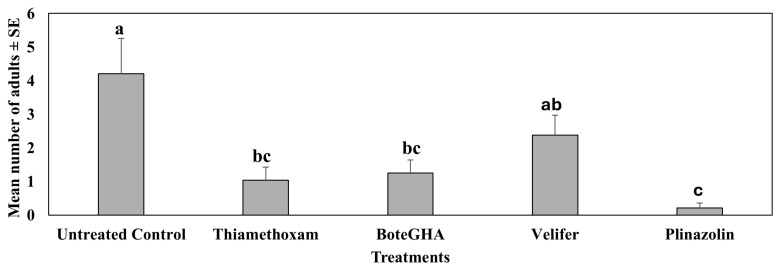
Mean number of adults on plants ± SE during the 6-week sampling period in the fifth study, spring 2023 (Dec 2022–Mar 2023). Means with the same letter are not statistically different according to Tukey’s HSD Test at *p* < 0.05.

The above study (fourth study), containing biorational insecticides, was repeated in spring 2023 (Dec 2022–Mar 2023) (fifth study) when all insecticide treatments, except velifer, significantly reduced pepper weevil adults as compared to the untreated control (Figure 3). However, velifer (ca. 38% reduction) did not differ statistically from boteGHA and thiamethoxam, whereas isocycloseram (Plinazolin) reduced ca. 91% of the adults compared to the untreated control. The mean number of adults differed significantly across dates (F_5,90_ = 20.91, *p* < 0.0001) and treatments (F_4,90_ = 18.12, *p* < 0.0001), and there were moderate interactions between dates and treatments (F_20,90_ = 1.71, *p* = 0.046).

### 4.2. Treatment Effect on Pepper Weevil-Infested Fruit

In the first study conducted in spring 2019 (Mar–Jun), insecticide-treated plants did not have any fallen infested fruits on the first sampling date (21 March). On the second sampling date (29 March), all treated plants had significantly fewer infested fruits than the untreated control. Similarly, all insecticide treatments had significantly fewer infested fruits on the remaining sampling dates (4 April, 12 April, 19 April, and 26 April) as compared to the untreated control. Overall, the untreated control had a significantly higher number of infested fruits compared to the rest of the treatments. (Table 5).

**Figure 4:**
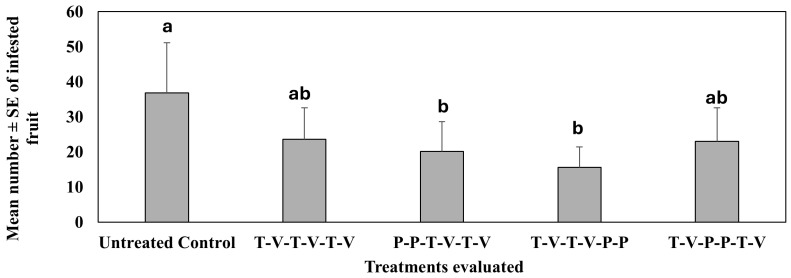
Mean number ± SE of infested fruit during the 6-week sampling period in the second study, spring 2021 (Mar–Jun). See Table 3 for the rotation schedule. T: Thiamethoxam, V: Vydate, P: Isocycloseram. Means with the same letter are not statistically different according to Tukey’s HSD Test at *p* < 0.05.

In the second study conducted in spring 2021 (Mar–Jun), using insecticides in rotation, the mean number of pepper weevil-infested jalapeño pepper fruits was significantly fewer in plots where isocycloseram was sprayed on the first and second spray dates (ca. 41%) and on the fifth and sixth spray dates (ca. 46%) as compared to the untreated control (Figure 4). When isocycloseram was applied on the third and fourth spray dates, the infested fruit reduction (38%) was not significantly different from the untreated control. However, there were no significant differences across all insecticide-treated plots. There were significant differences across dates (F_5,87_ = 303.28, *p* < 0.0001) across treatments (F_4,87_ = 5, *p* = 0.001), and there were significant interactions between dates and treatments (F_20,87_ = 2.40, *p* = 0.003).

**Figure 5:**
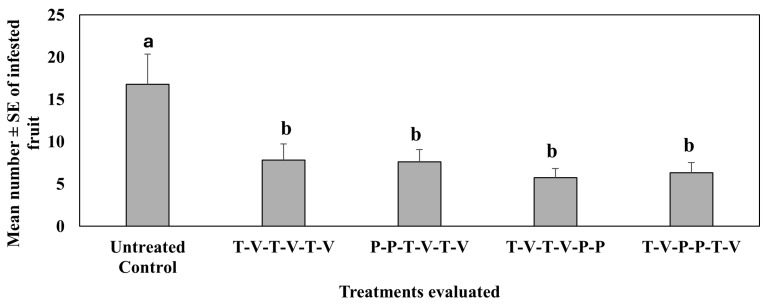
Mean number ± SE of infested fruit during the 6-week sampling period in the third study, spring 2022. See Table 3 for the rotation schedule. T: Thiamethoxam, V: Vydate, P: Isocycloseram. Means with the same letter are not statistically different according to Tukey’s HSD Test at *p* < 0.05.

In the third study (repetition of the second study), conducted in spring 2022 (Feb–Mar), significantly fewer infested fruit /treatment plots were recorded in all treated plots where isocycloseram was rotated back-to-back at different times of pepper plants’ growth, followed by or preceded by thiamethoxam and oxamyl (Figure 5). Similar results were recorded when only thiamethoxam was rotated with oxamyl. However, there were no significant differences across all treated plots.

There were significant differences across dates (F_6,102_ = 89.17, *p* < 0.0001) across treatments (F_4,102_ = 8.74, *p* < 0.001), and there were significant interactions between dates and treatments (F_24,102_ = 1.78, *p* = 0.03).

**Figure 6:**
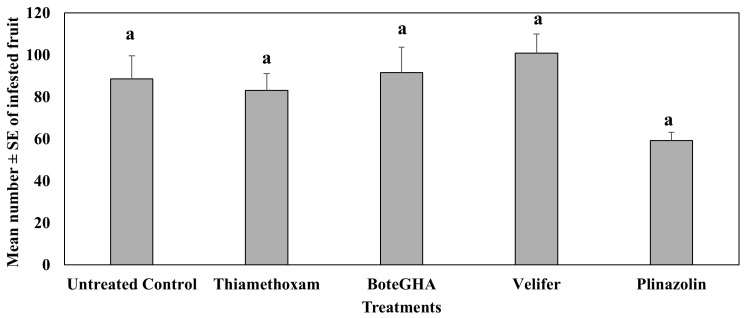
Mean number ± SE of infested fruits during the 6-week sampling period in the fourth study, spring 2022 (Mar–Jun). Means with the same letter are not statistically different according to Tukey’s HSD Test at *p* < 0.05.

In the fourth study conducted in spring 2022 (Mar–Jun), where *Beauveria bassiana-based* biorational insecticides were compared with conventional insecticides, isocycloseram (Plinazolin)-treated plants had a numerically lower number (ca. 37%) of infested jalapeño pepper fruit compared to the untreated control. When means were compared across all sampling dates, there were no significant differences in the mean number of infested fruits amongst treatments (F_4,132_ = 1.17, *p* = 0.33) (Figure 6).

**Figure 7:**
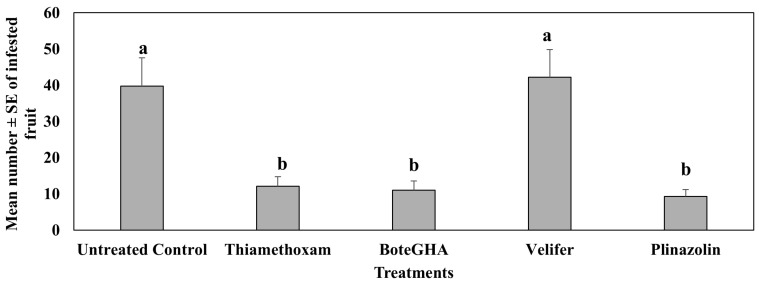
Mean number ± SE of infested fruits during the 6-week sampling period in the fifth study, spring 2023 (Dec 2022–Mar 2023). Means with the same letter are not statistically different according to Tukey’s HSD Test at *p* < 0.05.

In the fifth study in spring 2023 (Dec 22–Mar 2023; Figure 7), where the fourth study was repeated in 2023, all insecticide treatments, except velifer (*Beauveria bassiana* strain PPRI 5339), had significantly fewer pepper weevil-infested jalapeño pepper fruit than the untreated control (F_4,105_ = 33.67, *p* < 0.0001). The effect of *Beauveria bassiana* strain boteGHA in reducing pepper weevil-infested jalapeño (ca. 73%) did not differ from thiamethoxam (ca. 72.73%) and isocycloseram (Plinazolin) (ca. 72.32%). The mean number of infested fruits differed significantly across dates (F_6,105_ = 73.77), and there were moderate interactions between dates and treatments (F_24,105_ = 2.30, *p* = 0.020).

### 4.3. Marketable Yield

In the first study conducted in spring 2019 (Mar–Jun), thiamethoxam-treated plots had significantly higher yields than flupyradifurone and spirotetramat in rotation with flupyradifurone-treated plots. The marketable yield in spirotetramat-alone-treated plots did not differ from thiamethoxam-treated plots. Overall, insecticide-treated plants did not differ from the untreated control plants in the mean marketable yield (Table 6).

**Figure 8:**
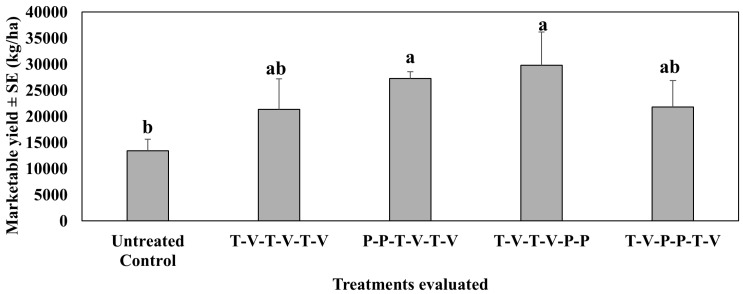
Marketable yield ± SE (kg/ha) during the 6-week sampling period in the second study, spring 2021 (Mar–Jun). See Table 2 for the rotation schedule. T: Thiamethoxam, V: Vydate, P: Isocycloseram. Means with the same letter are not statistically different according to Tukey’s HSD Test at *p* < 0.05.

In the second study conducted in 2021, where insecticides were rotated, all treated plants numerically or significantly increased marketable yields as compared to the untreated control (Figure 8). Marketable yield was significantly higher than the untreated control (F_4,12_ = 4.32, *p* = 0.02) when isocycloseram was applied two times back-to-back either at the beginning (first and second spray dates) or at the end (fifth and sixth spray dates). Mean marketable yield did not differ from the untreated control when isocycloseram was applied in the middle of the spray period back-to-back two times on the third and fourth spray dates. Thiamethoxam-oxamyl rotation at weekly intervals, a commonly used growers’ standard, did not differ from the untreated control in the mean marketable yield.

**Figure 9:**
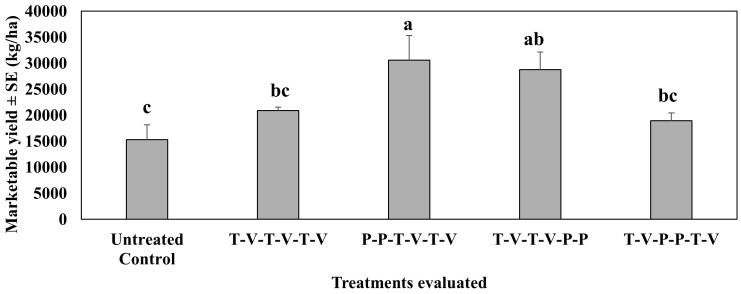
Marketable yield ± SE (kg/ha) during the 6-week sampling period in the third study, spring 2022 (Feb–May). See Table 3 for the rotation schedule. T: Thiamethoxam, V: Oxamyl, P: Isocycloseram. Means with the same letter are not statistically different according to Tukey’s HSD Test at *p* < 0.05.

In the third study, conducted in spring 2022 (Mar–Jun), repeating the treatments as in the first study, the mean marketable yield of jalapeño pepper in different treatment plots did not differ from that of the 2021 study, documenting significant marketable yield due to the appropriate placement of isocycloseram (Figure 9). Isocycloseram applied at the beginning or the end in two back-to-back applications provided a higher mean marketable yield than the untreated control. Mean marketable yield in plants treated with isocycloseram in the middle of the spray season (third and fourth spray dates) and thiamethoxam-oxamyl weekly for six weeks in a rotation program did not differ from the untreated control, although the yield was moderately higher than the untreated control. As a result, these two treatments did not differ significantly from the most effective two treatments, where isocycloseram was applied at the beginning or end of the spray season.

**Figure 10:**
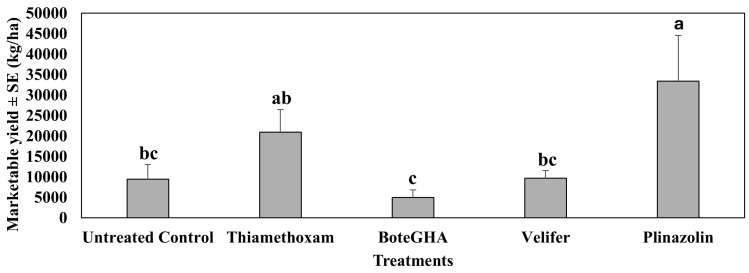
Marketable yield (kg/ha) ± SE during the 6-week sampling period in the fourth study, spring 2022 (Mar–Jun). Means with the same letter are not statistically different according to Tukey’s HSD Test at *p* < 0.05.

In the fourth study in spring 2022 (Mar–Jun), where biorational insecticides were compared with conventional insecticides, isocycloseram (Plinazolin) applied weekly for six weeks significantly increased the marketable yield of jalapeño pepper (F_4,12_ = 8.14, F = 0.0021) as compared to the untreated control (Figure 10). Thiamethoxam increased the marketable yield by 2x of the untreated control but did not statistically differ from the control. *Beauveria bassiana* strain BoteGHA-treated plants had the lowest marketable yield, followed by *Beauveria bassiana* strain PPRI 5339 (Velifer), and did not differ from the untreated control.

In the fifth study in spring 2023 (Dec 2022–Mar 2023, Figure 11), repeating the same treatment as in the fourth study of spring 2022, significantly higher marketable yield was recorded when jalapeño pepper plants were sprayed with isocycloseram (Plinazolin) and thiamethoxam (F_4,12_ = 9.27, *p* < 0.0012). The marketable yield from *Beauveria bassiana* strain PPRI 5339 (Velifer)-treated plants was higher than the untreated control, but not significantly different. *Beauveria bassiana* strain BoteGHA-treated plants had the lowest marketable yield, which was less than that of the untreated control.

## 5. Discussion

Development of a successful strategy for effective management of pepper weevils is challenging. Chemical insecticides are the primary management option for controlling insect pests. In this present study, we tested the efficacy of various chemical and biorational insecticides in managing pepper weevil to minimize resistance development against the most commonly used insecticides, thiamethoxam (Actara^®^) and oxamyl (Vydate L^®^). In the first study, spirotetramat (Movento^®^) and flupyradifurone (FPF, Sivanto^®^) were applied along with thiamethoxam (Actara^®^). Thiamethoxam (Actara^®^) is a xylem-systemic insecticide effective against beetles [32,33] and has been found to be effective in managing pepper weevil [23]. However, in the present study, thiamethoxam application did not show any significant reduction in pepper weevil as compared to the untreated control. This may be because thiamethoxam (Actara^®^) has been used for a long time to control this pest in the present area. Other possible explanations could be the concentration of the insecticide, prevailing temperature, and moisture at the individual adult’s microhabitat during the time of application. Table 7 shows the average temperature during the various growing seasons in this study, which could have impacted the effectiveness of the insecticides and the abundance of pepper weevils over time. Arthur et al. [32] found that parameters like concentration of thiamethoxam and temperature impacted the toxicity of this insecticide to stored product insects’ survival and mortality. However, Maienfisch et al. [34] reported promising performance of thiamethoxam against the Colorado potato beetle (*Leptinotarsa decemlineata* Say), flea beetles (*Aphthona flava*), and click beetles (Elaterid Spp.). Thiamethoxam was reported to cause mortality of five stored product beetle species after a short exposure [33]. This also has been observed by García-Nevárez [17], where malathion was found to be less effective in controlling the weevil in South-Central Chihuahua because of its long-term use. Similar findings were also observed by Avendaño et al. [24], where the pepper weevil population from one of the three locations (La Cruz de Elota) was found to be highly tolerant to thiamethoxam compared to the pepper weevil populations from the two other locations (Culiacan and El Rosario). Therefore, it cannot be ruled out that this weevil has undergone strong selection pressure by thiamethoxam (Actara^®^). The results of the inefficacy of movento and sivanto in controlling pepper weevil are from the earlier studies by Qureshi and Kostyk [35] and Seal et al. [5], where application of these insecticides did not cause a significant reduction in weevil population. This emphasizes the need to rotate insecticides with different modes of action to avoid high insecticide tolerance over time. However, sivanto prime 200 SL and Plinazolin SC400 reduced adult flea beetles significantly on cabbage [36]. Sivanto prime 200 SL at 10 fl oz/acre also reduced Egyptian alfalfa weevil larvae compared to the untreated check seven days after treatment application [37]. Sivanto prime and Plinazolin SC400 reduced alfalfa weevil larvae [38]. Movento was not as efficacious as other foliar insecticides evaluated against the Colorado potato beetle [39].

Therefore, we tested a novel insecticide, isocycloseram (Plinazolin), that works as a GABA receptor antagonist in insects [40]. The application of isocycloseram showed significantly better control of pepper weevil adults when rotated with thiamethoxam and oxamyl than the untreated control plots or just the plots where thiamethoxam and oxamyl were rotated. The results align with recent findings where isocycloseram has been found to be highly effective in managing coleopteran pests, alfalfa weevil [41], Colorado potato beetle [42], southern corn rootworm [43], cotton boll weevil [44], and clover seed weevil [45]. The difference in the mode of action of isocycloseram compared to the commonly used insecticides could be the reason behind the increased mortality. Therefore, the study identified a useful novel molecule for the management of pepper weevil, which may accelerate its use in pepper fields. When rotated with the few effective insecticides, it could potentially delay resistance development in pepper weevil.

Biorational insecticides can be used earlier in the season when the pepper weevil population is low and can also be used in combination with other integrated pest management strategies. Garcia-Nevarez et al. [17] reported that spinetoram and azadirachtin were not effective in reducing pepper weevil populations. From our study, the biorational insecticides were not efficient in reducing pepper weevil-infested fruits and increasing the marketable yield of jalapeño pepper. However, Labbé et al. [20] showed that kaolin clay and mineral oil reduced offspring weevil emergence by 59 and 54%, respectively, compared with untreated controls. Therefore, other molecules needed to be tested for the development of a successful IPM program for pepper weevil management. This will lead to the establishment of the best tools and practices for achieving year-round management of pepper weevil, as well as minimizing crop losses by this challenging pest in pepper fields.

Adjuvants, including Dyne-amic (containing methyl esters of C16 and C18 fatty acids, polyalkyleneoxide-modified polydimethylsiloxane, and alkylphenol ethoxylate), help to improve the efficacy of insecticides [46]. However, it has also been reported to have an inconsistent effect when mixed with insecticides. In a study conducted by Stanley [47], the adjuvants tested in his studies did not affect the efficacy of the insecticides significantly. Song et al. [48] also mentioned that the improper selection and inadequate use of adjuvant can lead to counterproductive effects on the crop as well as a waste of both adjuvant and insecticide. In the case of our study, we observed that the adjuvant, Dyne-amic, mixed with the insecticide boteGHA, showed negative effects on the fruit, leaves, and pepper yield. With regard to the mixing of adjuvants and other insecticides tested in our study, no phytotoxicity was observed.

The level of resistance or susceptibility of pepper weevils to insecticides varies depending on the amount and the number of times an insecticide is applied in a location over time [18]. Servin Villegas et al. [18] reported that pepper weevil was resistant to carbaryl, endosulfan, and methomyl in one of the three locations evaluated. Thiamethoxam is one of the efficacious insecticides for pepper weevil management. It is important to rotate it with insecticides with other modes of action to reduce the fast development of resistance [17]. Thiamethoxam was also effective in reducing the number of pepper weevils during 5 days of observation after application and was also used in a rotation study with other insecticides, including chlorpyrifos and spinetoram [17]. However, Avendaño et al. [24] reported that the pepper weevil populations from one of the three locations (La Cruz de Elota) evaluated were highly tolerant to thiamethoxam compared to the pepper weevil populations from the two other locations (Culiacan and El Rosario). This emphasizes the need to rotate insecticides with different modes of action to avoid high tolerance to insecticides over time. In our study, isocycloseram performed significantly better when rotated with thiamethoxam and oxamyl than the untreated control plots or just the plots where thiamethoxam and oxamyl were rotated.

The use of insecticides could be combined with other approaches, including the use of reflective mulch. Few studies have reported the additive effect of insecticides and reflective mulch. For example, the whitefly-transmitted virus of zucchini squash was suppressed in treatment plots with reflective mulch in combination with imidacloprid compared to plots with standard white mulch [49]. Combining the use of insecticidal sprays and reflective plastic mulch reduced the development of the whitefly-transmitted viral watermelon vine decline symptoms on fruits and plants [50]. Adeleye et al. [51] observed a reduction in the number of pepper weevil adults and infested pepper fruit when reflective silver on white mulch was combined with insecticides, thiamethoxam, and oxamyl.

In the future, another study that will compare insecticide treatments with and without an adjuvant should be conducted to evaluate treatment effect on pepper weevil. This will also determine if the adjuvants are helpful in increasing the efficacy of the insecticides used in this study for pepper weevil management.

## 6. Conclusions

Some of our studies reiterate the importance of rotating insecticides with different modes of action, as this helps to delay the fast development of resistance. From the results, we can conclude that isocycloseram, thiamethoxam, and oxamyl are efficient in reducing the pepper weevil population and increasing pepper yield. Therefore, isocycloseram can be used in rotation with thiamethoxam and oxamyl.

## Figures and Tables

**Figure 11:**
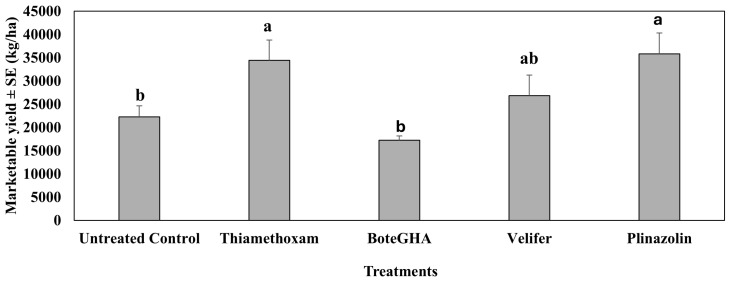
Marketable yield (kg/ha) ± SE during the 6-week sampling period in the fifth study. Spring 2023 (Dec 2022–Mar 2023). Means with the same letter are not statistically different according to Tukey’s HSD Test at *p* < 0.05.

**Table 1:** Various insecticide treatments used in the first study to suppress the pepper weevil.

Treatments	Insecticides	Rate [oz]/Acre (kg/ha)
1	Spirotetramat 240 SC + Dyne-amic	5.0 (0.35) + 0.25% *v*/*v*
2	Flupyradifurone + Dyne-amic	14.0 (0.98) + 0.25% *v*/*v*
3	Spirotetramat 240 SC + Dyne-amic rotated with Flupyradifurone HL + Dyne-amic	5.0 (0.35) + 0.25% *v*/*v* 14.0 (0.98) + 0.25% *v*/*v*
4	Thiamethoxam	4.0 (0.28)
5	Control	

**Table 2:** Insecticides used, application rate, and timing for the second, third, fourth, and fifth studies.

Insecticide	Product Name	Active Ingredient	Rate	Timing		Company
1	Control	-	-	-		
2	Actara	Thiamethoxam	5 oz/acre	Every 7 days	4A	Syngenta (Greensboro, NC, USA)
3	Vydate	Oxamyl	2 pint/acre	Every 7 days	1A	Dupont (Wilmington, DE, USA)
4	Plinazolin	Isocycloseram	3.1fl oz/A	Every 7 days	30	Syngenta (Greensboro, NC, USA)
5	Velifer	*Beauveria bassiana*	500 mL/L	Every 7 days	Omri listed	BASF (Florham Park, NJ, USA)
6	BoteGHA	*Beauveria bassiana* strain GHA	1 qt/acre	Every 4 days	Omri listed	Certis USA (Columbia, MD, USA)
	Dyne-amic	*Methylated seed oils*	0.25 *v*/*v*			Helena Agri-Enterprises, LLC (Collierville, TN, USA)

**Table 3:** Spray timing and application rotation of Plinazolin (isocycloseram) in the second and third studies.

Trt	Wk 1	Wk 2	Wk 3	Wk 4	Wk 5	Wk 6
1 W	Control	Control	Control	Control	Control	Control
2 R	Isocycloseram + Dyne-amic	Isocycloseram + Dyne-amic	Thiamethoxam	Oxamyl	Thiamethoxam	Oxamyl
3 B	Thiamethoxam	Oxamyl	Isocycloseram + Dyne-amic	Isocycloseram + Dyne-amic	Thiamethoxam	Oxamyl
4 G	Thiamethoxam	Oxamyl	Thiamethoxam	Oxamyl	Isocycloseram + Dyne-amic	Isocycloseram + Dyne-amic
5 Y	Thiamethoxam	Oxamyl	Thiamethoxam	Oxamyl	Thiamethoxam	Oxamyl

**Table 4:** Mean number of PW adults on different sampling dates treated with various insecticide treatments in the first study, spring 2019 (Mar–Jun).

Treatments	Rate [oz]/A	21 Mar	29 Mar	4 Apr	12 Apr	19 Apr	26 Apr
Spirotetramat 240 SC	5.0	0 a	0.00 b	0.00 b	0.10 ab	0.00 a	0.35 a
Flupyradifurone	14.0	0 a	0.00 b	0.00 b	0.10 ab	0.10 a	0.35 a
Spirotetramat 240 SC Rotated with Flupyradifurone HL	5.0 14.0	0 a	0.10 a	0.05 ab	0.35 a	0.10 a	0.25 a
Thiamethoxam	4.0	0 a	0.00 b	0.00 b	0.00 b	0.10 a	0.05 a
Control		0 a	0.00 b	0.15 a	0.25 ab	0.25 a	0.45 a

Means within a column followed by the same letter do not differ significantly (Waller–Duncan K-ratio *t*-test). Dyne-amic was added to all treatments at the rate of 0.25% *v*/*v* except Thiamethoxam.

**Table 5:** Mean number of pw-infested fallen fruit/treatment plot on different sampling dates treated with various insecticide treatments in the first study, spring 2019 (Mar–Jun).

Treatments	Rate [oz]/A	21 Mar	29 Mar	4 Apr	12 Apr	19 Apr	26 Apr
Spirotetramat 240 SC	5.0	0.00 b	0.25 b	0.00 b	0.20 b	0.15 c	0.30 b
Flupyradifurone	14.0	0.30 b	0.30 b	0.00 b	0.25 b	0.55 b	0.30 b
Spirotetramat 240 SC rotated with Flupyradifurone HL	5.0 14.0	0.00 b	0.30 b	0.10 b	0.25 b	0.10 c	0.40 b
Thiamethoxam	4.0	0.00 b	0.30 b	0.00 b	0.00 b	0.15 c	0.15 b
Control		0.95 a	1.50 a	1.45 a	1.65 a	2.15 a	2.00 a

Means within a column followed by the same letter do not differ significantly (Waller–Duncan K-ratio *t*-test). Dyne-Amic was added to all treatments at the rate of 0.25% *v*/*v* except Thiamethoxam.

**Table 6:** Mean weight (lbs.) of peppers harvested/plot sprayed with various insecticide treatments, spring 2019.

Treatments	Rate [oz]/A	Weight of Harvested Fruits/Plot (in lbs.)
Spirotetramat 240 SC	5.0	4.50 ab
Flupyradifurone HL	14.0	3.25 bc
Spirotetramat 240 SC rotated with Flupyradifurone HL	5.0 14.0	2.75 c
Thiamethoxam	4.0	5.18 a
Control		4.12 abc

Means within a column followed by the same letter do not differ significantly (Waller–Duncan K-ratio *t*-test).

**Table 7:** Monthly average temperature data (F) during the five study seasons.

Study/Monthly Average (F)	Month 1	Month 2	Month 3	Month 4	Average for Growing Season (F)
1	64.98 (Jan)	71.40 (Feb)	70.44 (Mar)	75.02 (Apr)	70.46
2	71.21 (Mar)	73.83 (Apr)	77.72 (May)	79.32 (Jun)	75.52
3	69.89 (Feb)	73.13 (Mar)	75.24 (Apr)	77.96 (May)	74.06
4	73.13 (Mar)	75.24 (Apr)	77.96 (May)	79.33 (Jun)	76.42
5	68.11 (Dec)	66.89 (Jan)	71.09 (Feb)	72.42 (Mar)	69.63

## Data Availability

The original contributions presented in this study are included in the article. Further inquiries can be directed to the corresponding author.
